# A method for experimental warming of developing tree seeds with a common garden demonstration of seedling responses

**DOI:** 10.1186/s13007-020-00700-7

**Published:** 2021-01-06

**Authors:** E. R. V. Moler, G. Page, L. Flores-Rentería, C. G. Garms, J. B. Hull, H. F. Cooper, J. Swenson, S. Perks, K. M. Waring, A. V. Whipple

**Affiliations:** 1 Department of Biological Sciences, Northern Arizona University, Flagstaff, AZ 86011 USA; 2 Department of Forest Ecosystems and Society, Oregon State University, Corvallis, OR 97331 USA; 3 Department of Biology, San Diego State University, San Diego, CA 92182 USA; 4 Forest Engineering, Resources & Management, Oregon State University, Corvallis, OR 97331 USA; 5 USDA Forest Service, Dorena Genetic Resource Center, Cottage Grove, OR 97424 USA; 6 School of Forestry, Northern Arizona University, Flagstaff, AZ 86011 USA; 7 Present Address: College of Natural Resources, Center for Forest Nursery and Seedling Research, University of Idaho, Moscow, ID 83843 USA

**Keywords:** *In-situ* seed cone warming, Temperature sensors, Seed development, Climate change, Forest trees, Cohen’s local *f*^2^ effect size

## Abstract

**Background:**

Forest dieback driven by rapid climate warming threatens ecosystems worldwide. The health of forested ecosystems depends on how tree species respond to warming during all life history stages. While it is known that seed development is temperature-sensitive, little is known about possible effects of climate warming on seed development and subsequent seedling performance. Exposure of seeds to high air temperatures may influence subsequent seedling performance negatively, though conversely, warming during seed development may aid acclimation of seedlings to subsequent thermal stress. Technical challenges associated with *in-situ* warming of developing tree seeds limit understanding of how tree species may respond to seed development in a warmer climate.

**Results:**

We developed and validated a simple method for passively warming seeds as they develop in tree canopies to enable controlled study of climate warming on seedling performance. We quantified thermal effects of the cone-warming method across individual pine trees and stands by measuring the air temperature surrounding seed cones using thermal loggers and the temperature of seed cone tissue using thermocouples. We then investigated seedling phenotypes in relation to the warming method through a common garden study. We assessed seedling morphology, physiology, and mycorrhizal nodulation in response to experimental cone-warming in 20 seed-source-tree canopies on the San Francisco Peaks in northern Arizona, USA. The warming method increased air temperature surrounding developing seed cones by 2.1 °C, a plausible increase in mean air temperature by 2050 under current climate projections. Notable effect sizes of cone-warming were detected for seedling root length, shoot length, and diameter at root collar using Cohen’s Local *f*^2^. Root length was affected most by cone-warming, but effect sizes of cone-warming on root length and diameter at root collar became negligible after the first year of growth. Cone-warming had small but significant effects on mycorrhizal fungal richness and seedling multispectral near-infrared indices indicative of plant health.

**Conclusions:**

The method was shown to reliably elevate the temperature surrounding seed cones and thereby facilitate experimental *in-situ* climate warming research on forest trees. The method was furthermore shown to influence plant traits that may affect seedling performance under climate warming.

## Introduction

Forest tree mortality related to global climate warming is occurring worldwide [1]. Reproductive processes involved in seed production in trees are affected both indirectly and directly by environmental perturbations including changes in temperature [2–4]. A lack of practical methods for warming the environment in which seeds develop, particularly for tree species, limits our understanding of the sensitivity of seed production to higher temperatures related to climate change.

In situ experimental warming treatments employ either active or passive warming systems [5]. Passive warming systems do not require supplemental energy, and instead reduce the loss of emitted longwave radiation by sheltering surfaces from boundary layer turbulence [6]. The thermal effect of warming treatments must be quantified carefully to account for differences among experimentally warmed microsites [7], and care must be taken to shield thermal loggers from direct shortwave radiation in order to accurately estimate warming effects [8].

Past heat exposure may predispose plants to adaptive responses to future episodes of heat exposure (i.e. conditioning [9]). Conditioned responses may be attributable to altered hormones, nutrients, antibodies, small RNAs, and epigenomic changes to gene expression that may persist in a lineage across generations [10]. In some cases, conditioning affords organisms a more rapid adjustment to prevailing environmental conditions [11], which may be crucial during vulnerable early life stages of plants [12].

Tree life history stages from reproduction through seedling establishment are vulnerable to abiotic stress related to climate warming [13]. High temperatures and drought can limit seed production [2, 14] and seedling establishment [15]. Temperature affects the production and quality of tree seeds in forests ranging from dry temperate [14] to subarctic regions [16]. Longer periods of seed development (e.g. > 2 years for many pine species) present more opportunities for suboptimal temperatures to reduce seed quality [4]. Heat experienced by parental plants and directly by seeds can reduce seed viability and seedling vigor [3, 9], and maladaptively affect progeny bud burst phenology and cold hardiness [17]. While effects of elevated temperature during tree seed development have been studied with clones in temperature-controlled greenhouses [18] and by inferring temperature differences during seed development based on provenance climates [19], there is a dearth of knowledge of the consequences of warming during seed development in ecologically-realistic settings. For instance, a study by Carneros et al. [17], which found differences in bud burst and cold hardiness of Norway spruce (*Picea abies*) grown at different temperatures, was conducted by producing genetic replicates by somatic embryogenesis under cold (18 °C) and warm (28 °C) greenhouse conditions. Such controlled studies stand to provide insights into the mechanisms by which seedlings may be affected by warming during seed development, but do not readily improve understanding of phenomena at the landscape level. A lack of seed warming studies conducted in ecological settings has hindered our ability to predict possible large-scale consequences of seed warming for forest function and species diversity.

Phenotypic trait responses to environmental conditions vary both across species and intraspecific ecotypes, and are constrained by covariance among traits [20, 21]. Accordingly, although less common, assessment of a broad range of plant traits can deepen insights into possible trait limitations and tradeoffs associated with plant responses to warming [9, 21]. For instance, a common garden study of Douglas-fir (*Pseudotsuga menziesii*) found that combined measures of drought and cold stress tolerance revealed trait covariance in relation to coupled abiotic stressors, suggesting tradeoffs in stress tolerance mechanisms [22]. In response to heat exposure, plants display altered tissue allocation (e.g. the proportion of resources invested in root versus shoot growth [23]), altered microbial community assemblages and function [24], and reflect modified profiles of near-infrared electromagnetic radiation [25].

We present a simple and effective method for in situ warming of seed cones during seed development using southwestern white pine (SWWP; *Pinus strobiformis*); a long-lived conifer found in a wide range of climatic conditions across the southwestern USA and western Mexico. The species is threatened by an exotic fungal pathogen [26], exhibits greater drought sensitivity than co-occurring ponderosa pine (*P. ponderosae* [27]), is sensitive to interspecific competition [28], and is expected to undergo extensive constriction and fragmentation of the species’ historical range in response to climate change [29].

This study addressed two objectives, including: (1) introduce and evaluate a method for warming seed cones during development, and (2) demonstrate the effect of the cone-warming method on SWWP seedlings grown in a common garden and assessed for changes in above- and below-ground traits (morphological, foliar spectra, and mycorrhizal fungal communities). To address objective (1), we developed a method for warming seed cones as they develop in tree canopies and evaluated the effect of the method by comparing temperatures achieved by the cone-warming treatment and control. We also assessed how well temperature data from ground-based weather stations and HOBO loggers in canopies estimated the temperature of seed cones during development. To address objective (2), we demonstrated the effect of our warming method by quantifying effect sizes of controlled cone-warming on above- and below-ground traits of *P. strobiformis* seedlings grown for four years in a common garden. We focused our common garden measures on three aspects of plant traits expected to influence plant performance as the climate warms: (1) plant morphology, (2) foliar spectra, and (3) mycorrhizal fungal colonization. We anticipate that our method for studying plant trait responses to cone-warming will help expedite discovery of heat-adapted seed sources.

## Methods

### Cone warming treatment and temperature measures

Over the course of three sequential growing seasons (2014, 2015, and 2016), cone-warming treatments were deployed in tree canopies to develop, evaluate, and refine the cone-warming method presented here. In each deployment year, SWWP seed cones were passively warmed during the periods of fertilization and seed maturation, i.e. the full final growing season in the 27-month seed cone production cycle [30]. During the 2014 deployment, bagging materials were compared for their ability to warm seed cones in canopies, methods were developed for quantifying the warming effect, and warmed and unwarmed (control) seeds were collected for use in a common garden demonstration of seedling responses. During the 2015 deployment, the best bagging material based on performance during the 2014 season was evaluated for the temperature effect achieved by the cone-warming treatment and control groups at five new stands. The 2016 deployment was conducted to compare the effect of the cone-warming treatment on the temperature of seed cone tissues and the air surrounding seed cones, and to determine whether seed cone temperatures could be reliably deduced from measures of air temperature in canopies and at the ground level.

**Fig. 1:**
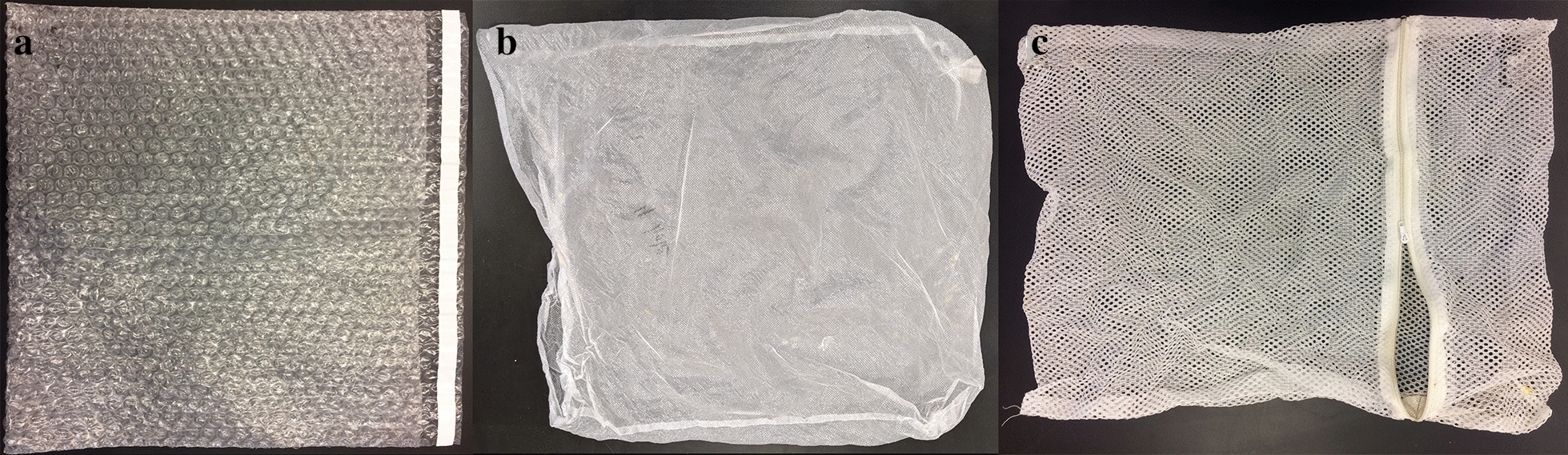
**a** Interior insulative bubble-wrap bag deployed in seed warming treatment. **b** Exterior low-airflow pollination bag deployed in seed warming treatment. **c** High-airflow mesh bag deployed in control treatment

**Fig. 2:**
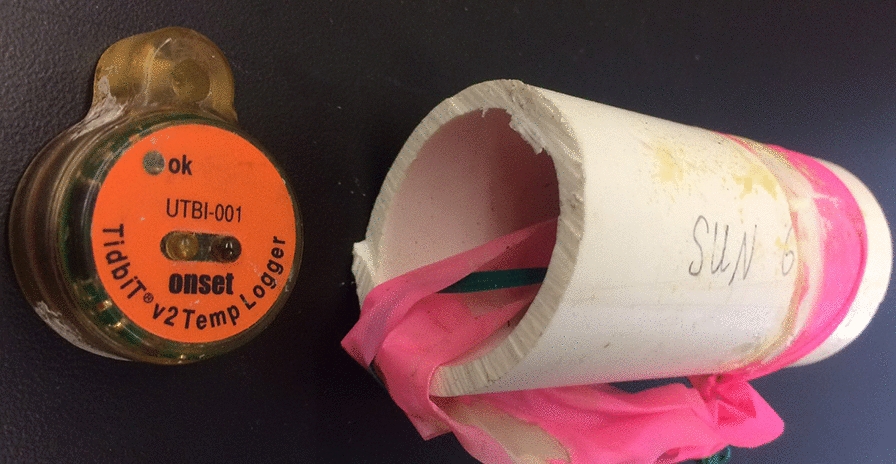
HOBO loggers were shielded from direct insolation by suspending HOBOs inside PVC tube segments

During the 2014 deployment (*n* = 20 trees in three stands throughout the San Francisco Peaks in northern Arizona), three to five controls and three to five cone-warming treatments were deployed in tree canopies. Each control and cone-warming treatment contained at least two seed cones. The cone-warming treatment in 2014 compared the efficacy of two materials for warming seeds: (1) a non-porous, insulative bag composed of translucent plastic bubble-wrap packaging material (Fig. 1a) inside of a low-airflow fine porous polyester pollination bag (Fig. 1b), and (2) a glassine bag. Bags were affixed to branches with Velcro tape. The warming effects of the two materials were not statistically different, and the bubble-wrap bagging material was preferred due to its greater durability. No bagging material was placed over control-group seed cones in 2014. Air temperature was measured inside and outside cone-warming treatments using HOBO loggers (ONSET^©^ HOBO V2 TidbiT Temperature Logger, Part # UTBI-001), suspended from the middle of a 10 cm long segment of white 2.54 cm diameter PVC tubing to shade loggers from direct insolation (Fig. 2), and hung from a branch with PVC tubes positioned laterally. In one of the three stands studied in 2014, two trees were affixed with one HOBO inside treatment bags (*n* = 2) and one HOBO outside treatment bags to record ambient air temperature (*n* = 2).

**Fig. 3:**
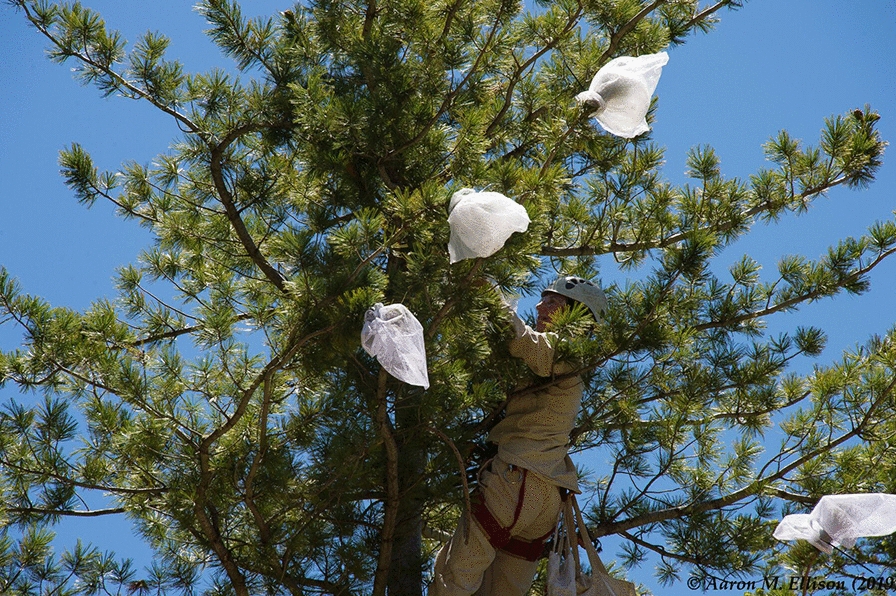
Warming treatment and control bags shown deployed in a seed source *P. strobiformis* tree. Photo copyright: Aaron Ellison

In 2015, cone-warming treatments and controls were deployed with HOBO loggers to quantify the effect of the treatment on air temperature at five additional stands (*n* = 1 treatment and *n* = 1 control per stand). The bubble-wrap material, which was found to be the most durable cone-warming bag type in the 2014 deployment, was the sole type of warming bag used in the 2015 deployment. Loss of treatment bags from branches during the 2014 deployment prompted us to use cable ties in 2015. Control and cone-warming treatment bags were loosely fitted around the cones, and bags were affixed to tree branches proximal to the cones by plastic cable ties placed over a ~ 5 cm segment of polyethylene foam pipe insulation used to increase the tree branch surface area affected by the cable tie (Fig. 3). Small branches and needles that spanned the pipe insulation barrier ensured channels for gas exchange. Whereas the 2014 deployment did not include a bag for the control, we included a control treatment bag from 2015 onward due to changing to the use of cable ties in order to ensure that the pressure that was exerted on branches was similar across cone-warming treatments and controls. The control treatment consisted of a high-airflow porous mesh nylon bag (Fig. 1c), while the cone-warming treatment consisted of the combined non-porous, insulative bubble-wrap packaging material (Fig. 1a) inside of a polyester pollination bag (Fig. 1b), as described above. Paired logged data (i.e. data from one cone-warming treatment and one control in a single tree) were retrieved from three of the five stands, whereas data from the fourth stand could only be retrieved from the control group and data from the fifth stand could only be retrieved from the cone-warming treatment due to loss of loggers during the course of the experiment (*n* treatment = 4, *n* control = 4).

We conducted a final experiment during the 2016 growth season to assess whether increased air temperatures inside cone-warming bags also increased the temperature of cone tissues. In contrast, only the temperature of air surrounding seed cones was measured during the 2014 and 2015 deployments, and not the temperature of seed cones themselves. In late May of 2016, cone-warming treatments and controls and two types of sensors were deployed in three *P. strobiformis* tree canopies 110 m from a weather station at Hart Prairie Preserve near Flagstaff, Arizona (35°21′06.0″N, 111°44′05.0″W). This experiment enabled evaluation of the effect of the cone-warming treatment on the temperature of seed cones using thermocouples, and to determine whether canopy air temperatures (measured with HOBO loggers) or air temperatures near the ground (measured with a thermistor 1.5 m aboveground) could be used to reliably estimate seed cone temperatures. In the canopies of three pines, three cone-warming treatment replicates and three control replicates were deployed. Each replicate contained at least two seed cones. A thermocouple was inserted into one cone within each control and cone-warming treatment bag to evaluate the effect of the cone-warming treatment on seed cone tissue (*n* treatment = 3, *n* control = 3). Thermocouple wires were inserted approximately 2 cm deep into seed cones. Each treatment and control bag in each tree contained one HOBO to evaluate the effect of the cone-warming treatment on air temperature within the bag, except in one of the three trees which received one HOBO in a cone-warming treatment. We obtained *n* treatment = 6 and *n* control = 4 HOBO data streams. Thermocouples logged temperature at five-minute intervals, and HOBOs logged temperature at hourly intervals. Temperature data were recorded from July–September.

### Cone warming treatment temperature analyses

The effect of the cone-warming treatment on cone tissue temperature was determined by fitting a linear model with the warmed cone temperature as the response variable and control cone temperature as the independent variable. We also compared the influence of the cone-warming treatment on the air temperature inside bags by fitting a linear model to HOBO logger data from inside the warming bag as the response variable and data from control bags as the independent variable. For the analysis of thermocouple data, measurements from the three cone-warming treatments and three control cones were averaged at each time point, then aggregated to daytime (7 am to 7 pm) and nighttime (7 pm to 7 am) average values. For HOBO logger measurements, replicates were averaged for each tree (*n* = 2 for the control, *n* = 3 for the cone-warming treatment), then an average value determined for all trees (*n* = 3). Values from HOBO loggers were also aggregated to daytime and nighttime averages. To compare measurements from thermocouples and HOBO loggers, we fit a linear model with warmed cone tissue temperature as the response variable and inside-bag air temperature as the independent variable. The passive warming treatment is most effective when incoming shortwave radiation inputs are greatest, hence models were fit separately for temperature values logged during day and night to more accurately quantify the daytime warming effect. We also calculated standard differences between average maximum monthly temperatures recorded in cone-warming treatment and control groups across all deployment years. To calculate standard treatment differences, we first estimated average maximum daily temperature per measurement and treatment type (e.g. thermocouple measurement in cone-warming treatment versus control) across all replicate measurements per year, calculated an average monthly maximum temperature from daily average maximum temperatures, and then calculated differences between the control group and warming group values.

### Common garden experiment

Seeds collected at the end of the 2014 cone-warming deployment were used in the common garden experiment. Following cone collection, cones were bench-dried in a greenhouse and extracted seeds were weighed in five replicated sets of ten seeds to estimate an average seed mass. Seeds were sown in the greenhouse in early October 2014 with subsequent greenhouse transplanting on November 18, 2014. Seeds were sown into labeled SC10 container growth tubes (Stuewe & Sons, Inc.; 3.8 cm diameter × 21 cm deep, 164 mL volume) in a completely randomized design across populations, genetic families, and cone-warming treatments. Seedling emergence occurred between 1 and 6 weeks following sowing. Seedlings were grown in the greenhouse for five months under ambient daylight conditions plus high pressure sodium lights to achieve a consistent 15 h. day: 9 h. night photoperiod. Seedlings were watered every other day and fertilized twice a week with 20-20-20 NPK fertilizer. Irrigation and fertilizer solutions were brought within a pH range of 5.5–6.2 using food grade phosphoric acid. Seedlings were placed outside of the greenhouse, and fertilization was ceased one month before outplanting to prepare seedlings for field conditions. Seedlings were then watered to keep the soil medium consistently moist. Replicates of each seedling experimental group (population, family, and cone-warming treatment) were planted into 1.2 × 1.2 m raised bed garden boxes constructed at the Arboretum at Flagstaff Southwest Experimental Garden Array site (35.1603° N, 111.7309° W). Soil medium in the boxes consisted of 50% Cornell soil mix (one-part sphagnum peat moss, one-part horticultural perlite, and one-part coarse vermiculite), and 50% volcanic cinders sourced from The Landscape Connection, Flagstaff. Just before planting, each raised garden bed was inoculated with one shovel-full of a mixture of soils gathered from all seed-source stands to include native soil microbes in the garden boxes. Eighty-one experimental seedlings were transplanted in a randomized design across both boxes in a 9 × 9 arrangement on June 6, 2015. Seedlings were spaced approximately 12 cm apart and competing vegetation was routinely removed to eliminate competition for light. Extra (i.e. non-experimental) seedlings were planted along box edges to buffer experimental seedlings from heat radiated by the sides of the raised-bed boxes during the day. Edge seedlings were clipped two years after planting to avoid unintended effects of belowground competition. An average of 8 seedlings were planted per each of the 20 seed source trees included in the common garden. Between one and 18 seedlings remained per seed source tree after the first year of growth. Each garden box was hand-watered using a spray wand fitted to a hose to apply 3.79 L of water every 7–10 days between the months of April and November.

Seedlings were grown for four summers until harvesting during the spring of the fifth growth season, on May 2, 2019. Traits measured in the common garden included (1) plant growth above-ground (measured annually) and below-ground (measured once during transplanting and once post-harvest), (2) multispectral and thermal indices via an unpiloted aircraft system (UAS) measured during the summer in 2017 and 2018, as in [31], and (3) morphotypic mycorrhizal nodulation (measured post-harvest in 2019). Plant growth traits included plant height measured as the distance from soil level to the top of the top-most bud on the central stem, diameter at root collar (DRC) measured as seedling stem diameter at soil-level, full shoot length measured as the distance from root collar to the top-most bud, full root length measured during transplanting to raised-bed garden boxes before the first summer of growth, root and shoot dry-mass measured post-harvest, and dates of bud development. Calculated plant growth traits included mean annual height and DRC growth increments (mean change (∆) in measure each year for both height and DRC), root-to-shoot length and mass (root measure divided by shoot measure, completed for both length and mass-based measures), yearly slenderness (shoot length divided by DRC), and full ∆ height and ∆ DRC (final measure minus initial measure, divided by initial measure). Multispectral and thermal infrared sensors carried by UAS recorded spectra at one timepoint at midday on May 18, 2017 and again at midday on June 2, 2018. Near infra-red spectra were used to estimate seedling crown temperatures, corresponding leaf-to-air temperature differences, and spectral indices indicative of plant health including the normalized difference vegetation index (NDVI), green NDVI (GNDVI), normalized difference red edge index (NDRE), triangular greenness index (TGI), and green–red vegetation index (GRVI). Post-harvest and before roots were dried, mycorrhizal fungi on seedling roots were assessed to the level of morphotype to determine whether cone-warming affected mycorrhizal assemblages. Mycorrhizal assemblages can affect plant performance [32], and mycorrhizal fungal species richness can be estimated by assessing mycorrhizal morphotypes [33]. Percent ectomycorrhizal fungal (EMF) colonization and EMF diversity were estimated on up to 100 root tips per seedling, noting (1) dead root tips, (2) live root tips, (3) dead EMF tips, and (4) live EMF tips. Each living EMF tip was assigned a morphotype designation based on color, texture, shape, and external hyphal characteristics following [33].

### Common garden statistical analyses

Multivariate and univariate models were used to investigate statistical relationships between the cone-warming treatment and response variables. Effect sizes of cone-warming on responses were then estimated as described below. All analyses were conducted in R (version 3.5.1, R Core Team 2018). Seedlings in the common garden demonstration from each type of cone-warming bag (glassine versus plastic bubble-wrap packaging material, both inside of a polyester pollination bag) were treated the same because there was no statistically significant difference between the effect of the two types of cone-warming bags on seedling traits. Multivariate models were built using both principal component analysis (PCA; via the function *prcomp*) and permutational multivariate analysis of variance (PERMANOVA; via the function *adonis*) separately for the following three categories of response variables: (1) plant growth traits including bud phenology, (2) foliar spectra, and (3) mycorrhizal assemblages. The first principal component of the PCA generated from variables, belonging to one response category at a time, was used as the response in linear mixed effect models. Next, PERMANOVAs were executed using Euclidean distance matrices composed of aggregated response variables for each of the three response categories (plant growth, spectra, and mycorrhizae), separately, specifying seed source tree as a random effect. Univariate linear mixed effect models were also fitted for all response variables, specifying seed source tree nested within stand, and raised-bed box (where models would allow), as random effects using functions from the R package *lme4*. In both multivariate and univariate models, seed mass was tested for inclusion as a covariate via AIC comparisons. Models with the smallest AIC were favored, and when models competed with AIC values within 2 AIC units of the smallest AIC, the simplest model structure with the least predictors was selected for subsequent ANOVAs. Satterthwaite approximation of denominator degrees of freedom was specified for all omnibus F-tests of fixed effects as well as type III sum of square ANOVAs for models that included interactions between seed mass and warming treatment. Type II sum of square ANOVAs were specified for models that included seed mass as an added covariate. The magnitude of the variance explained by the cone-warming fixed effect was estimated by Cohen’s Local *f*^2^, which is suitable for use with mixed models for which denominator degrees of freedom must be approximated, and is suitable for use with unbalanced experimental designs [34, 35]. Input for the calculation of Cohen’s Local *f*^2^ includes marginal R^2^ goodness-of-fit values both from models with and without the factor of interest, as follows:

$$f^{2} = \frac{{R_{with}^{2} - R_{without}^{2} }}{{1 - R_{with}^{2} }}$$

$R_{with}^{2}$ refers to the marginal coefficient of determination from a model containing a fixed factor of interest, and $R_{without}^{2}$ refers to the marginal coefficient of determination from the same model with the fixed factor of interest removed. For instance, in this study the warming treatment was present in the $R_{with}^{2}$ model and omitted from the $R_{without}^{2}$ model. Cohen’s Local *f*^2^ effect sizes ≥ 0.02, ≥ 0.15, and ≥ 0.35 are respectively considered small, medium, and large [35, 36]. Code and data related to this work are accessible through the Knowledge Network for Biocomplexity.

## Results

### Verification of cone-warming method

**Fig. 4:**
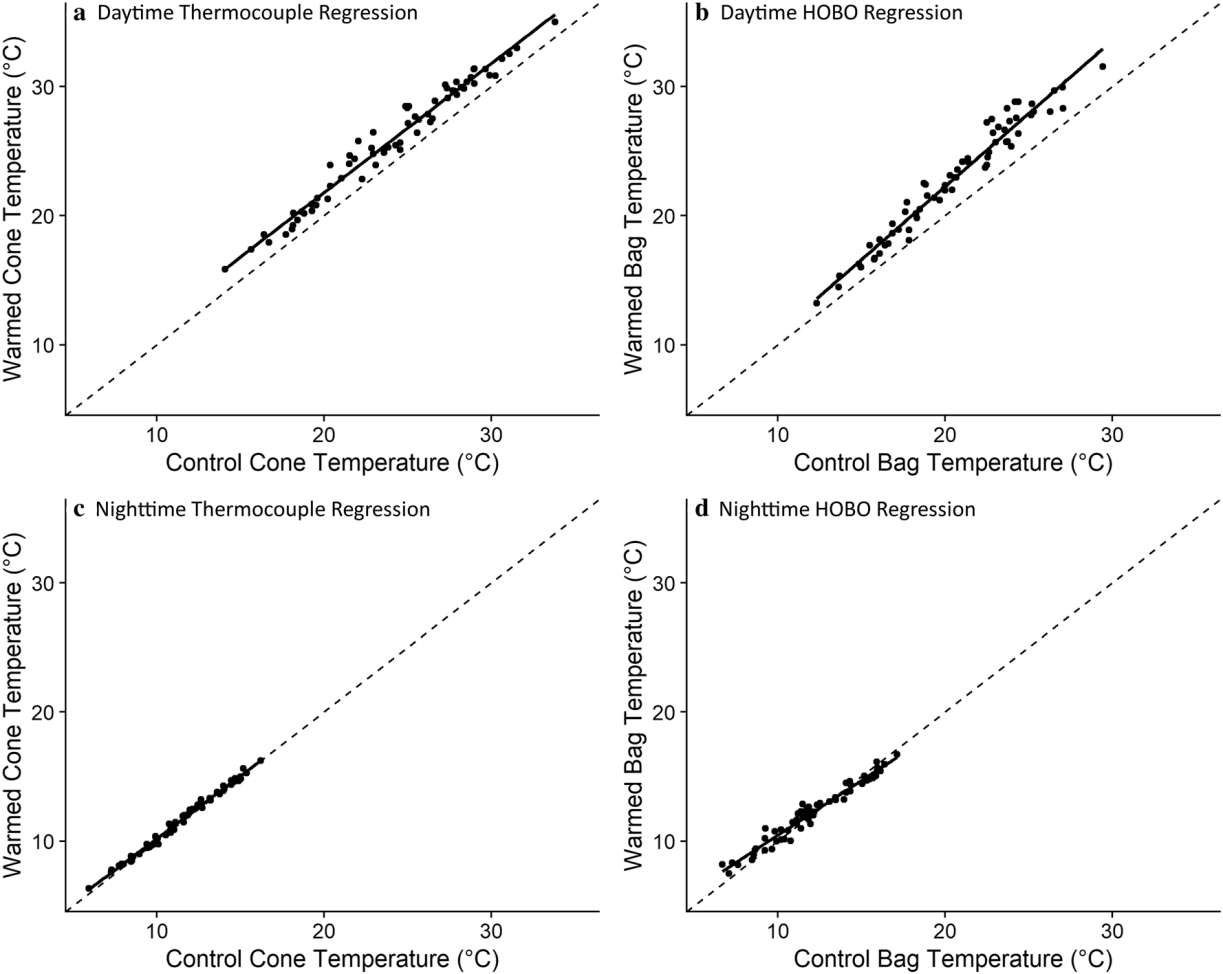
Comparisons of mean daytime and nighttime control and warmed treatments of cone and bag temperatures from 2016 thermal data. **a** daytime thermocouple and **b** daytime HOBO measurements. **c** Nighttime thermocoupleand **d** nighttime HOBO measurements. Filled black circles represent the mean daytime (7 am to 7 pm) or nighttime value each day from one tree. Dashed lines represent theoretical 1:1 relationships between data from control and warmed groups. Solid lines are linear model fits to the regression of temperature data from warmed versus control treatments

**Fig. 5:**
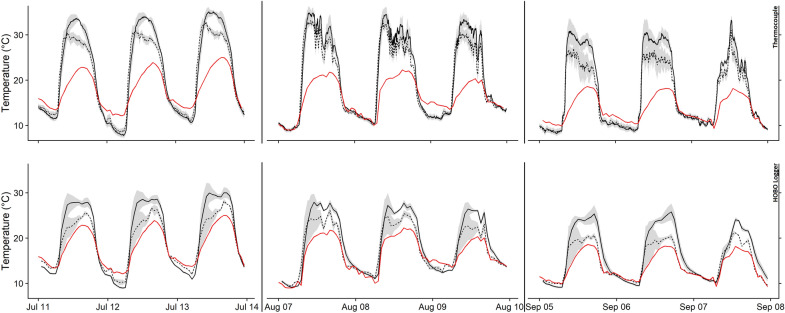
Daily Temperature Fluctuations by Logger Device. Hart Prairie temperatures for three-day periods in July, August and September 2016. The top three panels show data from thermocouples (measurements recorded every five minutes). The bottom three panels show data from HOBO loggers (measurements recorded once hourly). Black line shows data from the cone-warming treatment, dashed line shows data from the control, and red line is air temperature 1.5 m from the ground from a nearby weather station away from the forest overstory. Gray shading is the upper and lower bounds of the standard error range relative to the mean for each time point. Data represent averaged values across trees from which a standard error was calculated from averaged replicates within trees. Each line represents one data stream from a tree/treatment combination

Across all deployment years, temperature differences between the cone-warming treatment and control varied temporally, with the greatest increase in temperature due to the warming treatment recorded during the early summer (Table 1). During the 2016 growing season at Hart Prairie, thermocouples inside cones measured a statistically significant increase in daytime temperature of 0.9 °C in cone-warming treatments compared to controls (*t*_1,64_ = 45.4, *p* < 0.001; Fig. 4a). In the same experiment, HOBO loggers inside cone-warming bags measured a statistically significant increase in daytime mean air temperature of 2.1 °C in cone-warming treatments compared to controls (*t*_1,64_ = 37.3, *p* < 0.001; Fig. 4b). No significant cone-warming treatment effect was observed in data recorded at night (Figs. 4c, d). A linear model of within-cone temperature as a function of air temperature, with sensor type as an additive covariate, showed that daytime mean thermocouple measurements were 2.92 °C warmer than daytime HOBO measurements at the same air temperature. This standard offset value, which reflects temperature differences recorded by HOBO loggers and thermocouples inside cones, both inside cone-warming bags, allows us to predict cone interior temperature (as measured with thermocouples) based on air temperatures surrounding cones recorded by HOBO loggers inside cone-warming treatment bags. However, differences between temperatures measured by thermocouples inside cones and air temperatures measured by HOBO data loggers varied over the diurnal cycle and across months (Fig. 5). Temporal variation in temperature stability was also observed. For instance, during the North American monsoon, which affects the study region primarily during August and September, thermal variation increases coincide with increased daytime cloud cover, precipitation, and humidity (Fig. 5). Inconsistent differences between temperatures measured at the weather station and inside cone-warming treatments precluded calculation of a standard offset between those measurement locations. However, average daily temperatures recorded from a thermistor at 1.5 m above the ground surface at the weather station were always lower than those measured in canopies (Fig. 5), likely due to the combined effect of decreased albedo and increased boundary layer imposed by the canopy. During the period of June 28 to September 9th, 2016, mean daily air temperatures measured at the weather station were on average 2.6 °C cooler than air temperatures recorded in control-treatments deployed in nearby canopies. Average daily maximum values for 2016 HOBO logger data at Hart Prairie in July, August, and September were 40.5, 32.4, and 30.4 °C, respectively. Average daily maximum values for 2016 thermocouple data at Hart Prairie in July, August, and September were 36.0, 34.7, and 34.4 °C, respectively.

**Table 1:** Mean monthly daytime temperature increases (°C) of cone-warming treatment over controls as measured by HOBO loggers

	May	June	July	August	September
2014	3.30	4.06	2.63	2.50	NA
2015	5.35	3.70	3.47	2.79	NA
2016	NA	1.30	3.03	1.81	1.19

### Common garden experiment

**Fig. 6:**
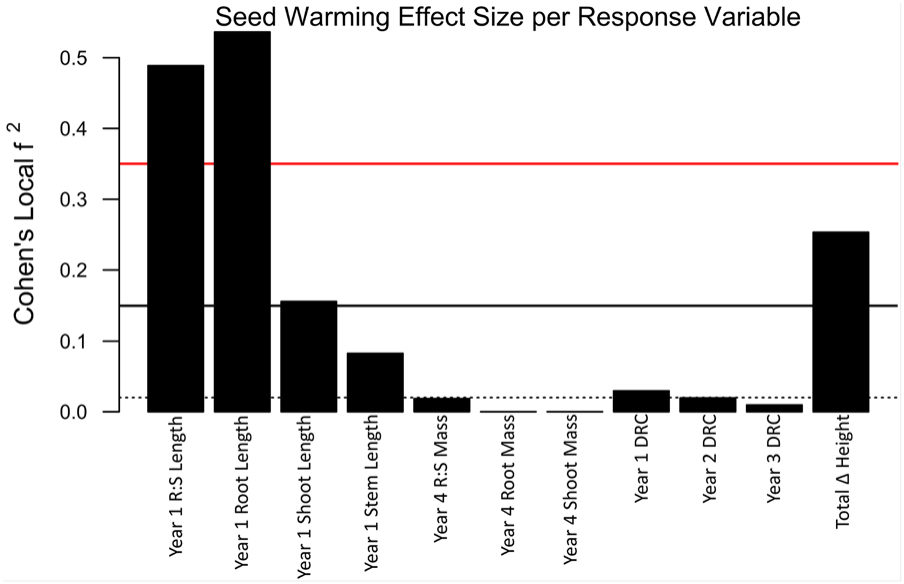
Effect sizes of cone-warming upon 11 response variables. Bar heights below the black dotted horizontal line at y = 0.02 are negligible effects. Bar heights between the black dotted and black solid horizontal lines are small but notable effects. Bar heights between the solid black horizontal line and solid red horizonal line are medium effects. Bar heights above the solid red horizontal line are large effects. 1st year of growth = 2015, 4th year of growth = 2018. 4th year growth measurements were taken during the spring prior to the beginning of the 5th growth year

Cone-warming produced notable effect sizes (i.e. Cohen’s Local *f*^2^ ≥ 0.02) for 1st year root length, 1st year root:shoot length, 1st year shoot length, 1st year stem length, 2nd year DRC, and total standard ∆ height (Fig. 6). Statistically significant interactions between cone-warming and seed mass showed that warming related to an increase of 0.3 cm in 1st year root length ($X_{1}^{2}$ = 4.0, *p* = 0.045), an increase of mycorrhizal morphotype richness from 3 to 3.2 ($X_{1}^{2}$ = 6.4, *p* = 0.01), and the PC1 composite of spectra measured in 2018 ($X_{1}^{2}$ = 6.8, *p* = 0.01). PC1 of 2018 spectra explained 77.9% of variation in spectral data. TGI accounted for the largest eigenvalue of PC1, and positive values of PC1 corresponded to larger values of TGI. While individual spectral values did not vary significantly across warming treatments, NDVI values measured in 2017 (mean = 0.37, SD = 0.05) were on average greater than those measured in 2018 (mean = 0.29, SD = 0.09). First-year root length was the only statistically significant response to cone-warming that also showed a notable effect size (Fig. 6**,** Table 2). While significant interactions between seed mass and cone-warming influenced mean values of multiple response variables, cone-warming did not affect seed mass directly (mean warmed seed mass = 1.67 g, SD = 0.32; mean control seed mass = 1.63 g, SD = 0.32).

**Table 2:** Summary statistics of response variables tested shown here with largest effect sizes at top and diminishing effect sizes toward bottom of table

Response	Treatment	Estimated mean	Lower 95% CI	Upper 95% CI	Cohen's Local *f*^2^	Seed warming *p* value
1st year root length	Control	19.7	18.9	20.5	0.536	0.03*
	Warmed	20.0	19.2	20.8		
1st year Root:Shoot length	Control	6.3	5.6	7.0	0.489	0.36
	Warmed	6.5	5.8	7.2		
Standard height change	Control	6.1	4.9	7.3	0.253	0.15
	Warmed	5.1	3.9	6.4		
Cotyledon No.	Control	11.5	10.4	12.5	0.171	0.76
	Warmed	11.5	10.4	12.6		
1st year shoot length	Control	3.3	2.7	4.0	0.155	0.58
	Warmed	3.3	2.6	3.9		
Phenology PC1	Control	0.7	0.4	1.0	0.107	0.13
	Warmed	1.0	0.7	0.0		
1st year stem length	Control	2.2	1.2	3.1	0.083	0.76
	Warmed	2.1	1.1	3.2		
Early needle length	Control	2.5	2.1	2.8	0.080	0.60
	Warmed	2.5	2.2	2.9		
Morphology PC1	Control	0.8	− 0.4	1.9	0.060	0.96
	Warmed	− 0.6	− 1.7	0.5		
2016 DRC	Control	3.8	3.5	4.2	0.030	0.62
	Warmed	4.3	4.0	4.6		
2017 DRC	Control	4.1	3.9	4.2	0.020	0.43
	Warmed	4.1	4.0	4.3		
4th year (harvest) root mass	Control	4.0	2.6	5.4	0.020	0.50
	Warmed	4.0	2.5	5.5		
4th year (harvest) Root:Shoot mass	Control	0.6	0.5	0.8	0.019	0.78
	Warmed	0.6	0.5	0.8		
2018 height	Control	13.5	11.5	15.5	0.015	0.89
	Warmed	13.4	11.3	15.6		
2017 height	Control	9.5	8.9	10.0	0.015	0.41
	Warmed	9.4	8.9	9.9		
Mycorrhizal richness	Control	3.0	2.7	3.4	0.012	0.02*
	Warmed	3.2	2.9	3.5		
2018 DRC	Control	5.4	4.9	5.9	0.010	0.22
	Warmed	5.6	5.1	6.1		
2018 spectra PC1	Control	1.0	0.7	1.2	0.008	0.01*
	Warmed	0.9	0.6	1.2		
2016 height	Control	8.6	7.2	10.0	0.004	0.67
	Warmed	9.2	8.0	10.5		
4th year (harvest) shoot mass	Control	6.5	5.2	7.9	0.004	0.35
	Warmed	6.6	5.1	8.0		
2017 spectra PC1	Control	− 0.1	− 2.2	2.0	0.001	0.58
	Warmed	0.1	− 2.4	2.6		

*Appears beside p-values for models that also showed significant interactions between cone-warming treatment and seed mass

## Discussion

It remains to be seen whether warmer future environmental conditions will influence forest regeneration through effects on the process of seed development*.* The method introduced here reliably increases mean air temperatures surrounding seed cones to an extent approximately matching the minimum mean air temperature rise expected in the USA by the year 2050 (assuming relative concentration pathways ≥ 4.5 [37]), and produces notable effects on seedling morphology. In our common garden demonstration, we found large effect sizes of cone-warming on morphological plant traits, but that those effects may be short-lived. We found a large effect size of cone-warming on root tissue allocation following eight months of growth in a greenhouse before seedlings were transplanted into common gardens. After four summers in the common gardens, however, the effect disappeared. Relatedly, the effect size of cone-warming on DRC, which is an indirect measure of belowground plant tissue allocation [38], also decreased with each year past the cone-warming treatment. Mortality of long-lived plant species is most likely to occur during the seedling stage soon after germination [39], and this is partly attributable to the small volume of seedling roots [40]. Thus, even short-lived increases in root tissue allocation in response to seed development under warmer conditions could improve seedling survival if warm conditions during seed development correlate with warm, dry conditions during early seedling growth.

Multispectral indices, particularly NDVI and TGI, provide useful indicators of plant health [25], and mycorrhizal communities influence plant water relations [41], but it is unclear why either would vary based on cone-warming treatments unless warming induced alterations in developmental pathways that persist through the transition from the zygote stage to the sporophyte stage. The 2018 PC1 of multispectral indices was significantly reduced by cone-warming. TGI, which indicates leaf chlorophyll content [42], was the spectral index with the largest eigenvalue of PC1, and this indicates that cone-warming may have resulted in deleterious effects on leaf phytochemistry. However, we emphasize that the statistical effect size was negligible, and that while PCA1 of multispectral indices differed significantly across cone-warming versus control treatments, individual indices did not. While bud phenology did not respond to cone-warming in our study, Johnsen et al. [18] found that bud phenology responded significantly to cone-warming in Norway spruce. This discrepancy highlights the need to evaluate the ecological relevance of notable trends detected in tightly controlled studies, such as that of Johnsen et al. [18] across species and in natural settings, as the method introduced here enables.

We did not compare relative humidity in warming versus control bags, which, all else being equal, would tend to decrease as temperature increases [43]. But we included as little mass as possible of non-cone plant tissue in warming bags to minimize accumulation of respired water vapor. We also do not expect that possible differences in light intensity between control and warming treatments would have influenced seed or seedling traits because pine seed-cones are composed of dead cells during the final summer of seed development [44], which is the only period during which our warming treatments were deployed. Thus, it is unlikely that a biological mechanism capable of detecting and responding to altered photosynthetic photon flux density occurs in mature cones.

There is a dire need for a more complete understanding of how climate change will affect the reproductive biology of forest trees and subsequent performance of seedlings. The method presented here provides a means for addressing a crucial knowledge gap concerning the effect of elevated air temperature during seed development on seed and seedling traits that influence forest regeneration. There is no clear consensus regarding how seed traits may respond to warming, though it has been argued that seed trait responses to climate warming are likely to vary by species [13]. For instance, Dewan et al. [19] reported reduced seed germination success of seeds from maternal trees grown under warmer conditions, while Alexander and Wulff [45] reported reduced germination success and altered phenology for *Plantago lanceolata* seeds produced by F_0_ and F_1_ plants following warming of the F_0_ generation environment. In contrast to these examples of warming negatively influencing seeds or seedlings, Scotch thistle (*Onopordum acanthium*) was reported to show increased germination for seeds from warmer maternal environments [46]. Studies of *Arabidopsis thaliana* demonstrated that warming of parental plants during flowering and seed development resulted in the expression of traits in progeny that may confer greater survival and fitness under elevated temperatures, and this effect related to increased nitrogen content in seeds from warmed parent plants [47]. While there are various mechanisms by which temperature may directly affect seed development [3, 4], the method presented here does not account for ways in which warming whole parental plants may affect seed and subsequent seedling performance, and this may represent a limitation of the method [48].

## Conclusion

The accessible, inexpensive method presented here can be used to warm reproductive structures in situ for virtually any type of land-plant, including forest trees. The broad applicability and simplicity of this method should facilitate a better understanding of the consequences of seed development under a warming climate for seed and seedling performance in multiple species and climate futures (e.g. RCP4.5—RCP8.5). We also anticipate that the method and demonstration presented here will lead to approaches for detecting genetic material with desirable climate-hardy traits.

## Data Availability

Upon publication, datasets used in this manuscript will be made available through the online Knowledge Network for Biocomplexity. The corresponding author will also provide datasets upon request.
